# Supplementary material for: Developing a Tanshinone IIA Memetic by Targeting MIOS to Regulate mTORC1 and Autophagy in Glioblastoma
Source: Int J Mol Sci. 2024 Jun 14;25(12):6586. doi: 10.3390/ijms25126586 (PMC11204349; doi:10.3390/ijms25126586)
Supplement: Supplementary file 1 [file ijms-25-06586-s001.zip › ijms-2974058-supplementary.pdf]

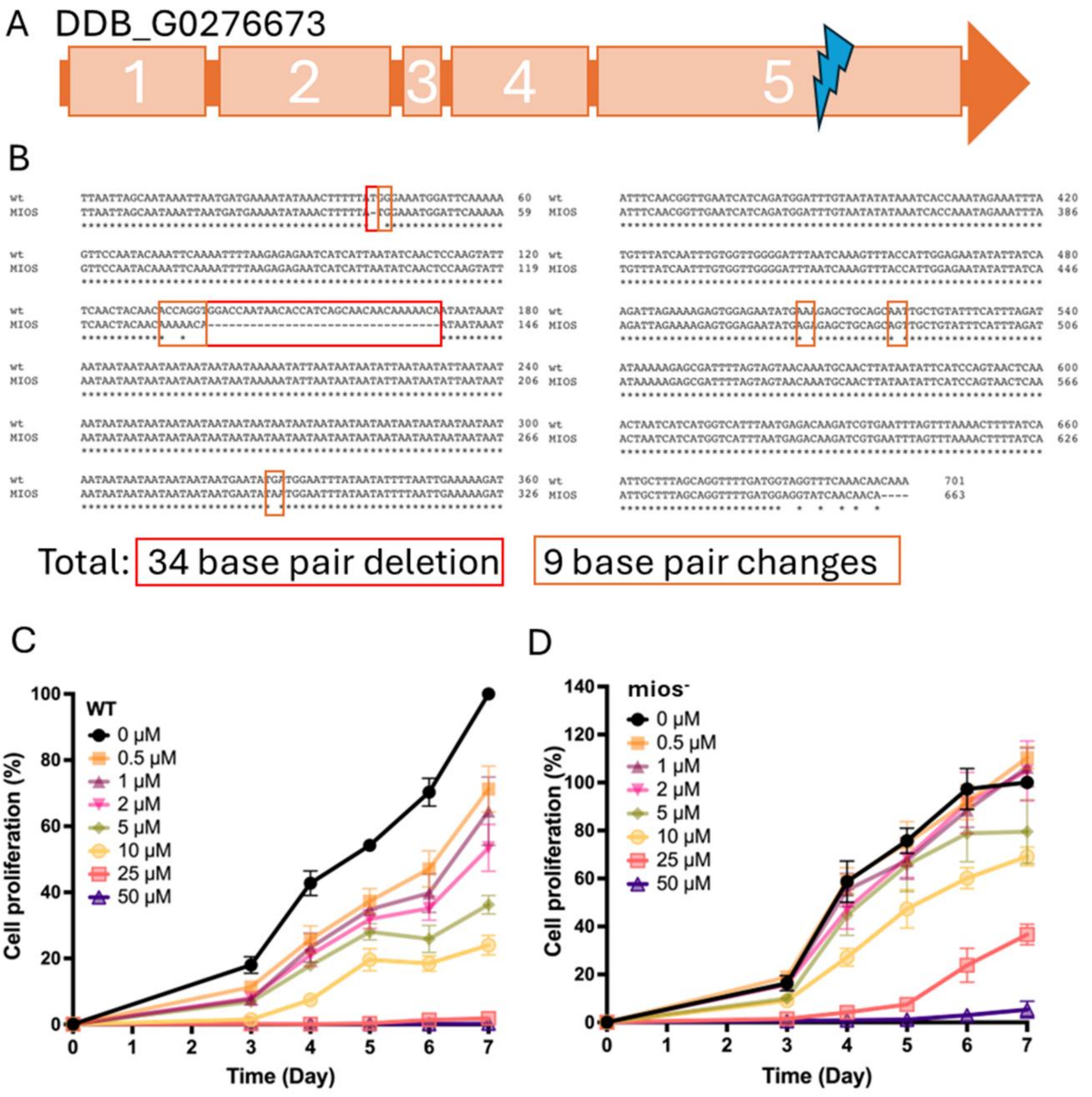

Figure S1: Development of the *D. discoideum mios*$^{-}$ CRISPR mutant. A. Shows the mios gene with 5 exons, where exon 5 was targeted by CRISPR techniques to render the gene inactive. B. The genomic sequence of a potential mutant was screened against the sequence of the wild type (Ax3) parent cell line showing that the mutant had a total of 34 base pairs deletion and 9 base pair changes (coloured boxes). C. The wild type and D. mios^{-} mutant cells were analysed in a cell proliferation assay following exposure to a range of T2A concentrations (0.5-50 μM) where mutant cells showed partial resistance to T2A, for example, with 0.5 μM T2A reducing cell proliferation by 25 % in wild type cells which is not observed in the mios^{-} cells.

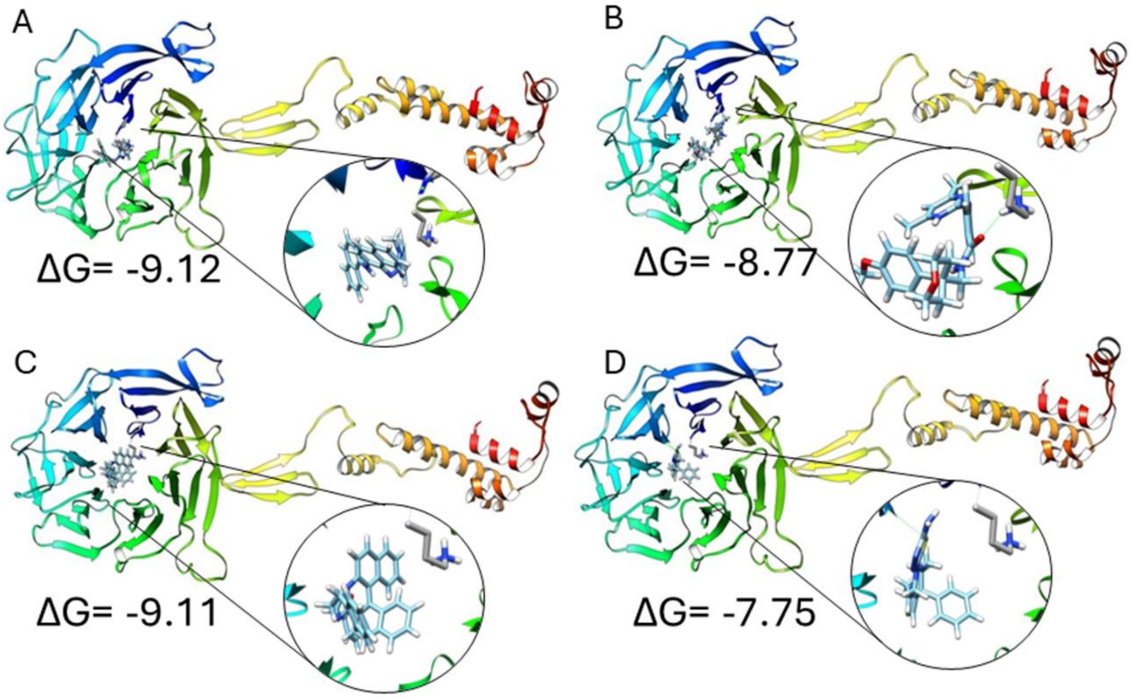

Figure S2: Computational analysis of the binding of other Mi compounds to human MIOS protein. A. Docking between Mi2 and the human MIOS protein shows likely binding to the interior of the β propeller structure with a ΔG value of –9.12 kcal/mol. Similar docking locations are predicted for: B. Mi4 with a ΔG value of –8.77 kcal/mol; C. Mi10 with a predicted ΔG value of –9.11 kcal/mol; and D. Mi20 with a predicted ΔG value of –7.75 kcal/mol.

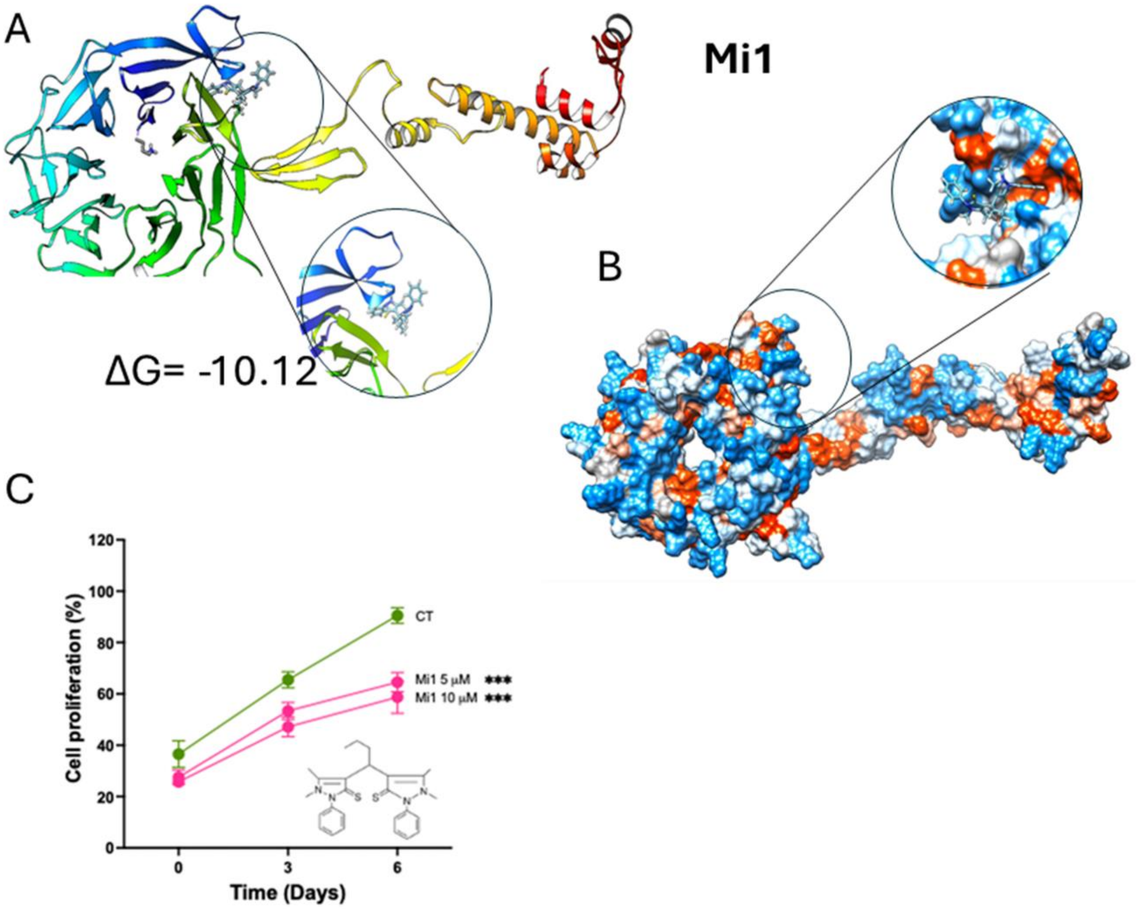

Figure S3: Mi1 inhibits cell proliferation of GL261 cells. A The schematic of the human MIOS protein where the Mi1 compound was docked, showing a binding site on the exterior region of the β-propeller structure with a delta G value of –10.12 kcal/mol. B. The hydrophobicity space filling model of the human MIOS protein with Mi1 showing a cleft in the structure within which Mi1 fits. C. Cell proliferation analysis over 6-days showed Mi1 treatment at both 5 and 10 μM significantly inhibited cell proliferation by 38 and 42 % respectively. All data shown ± SEM ***= $p < 0.0005$.

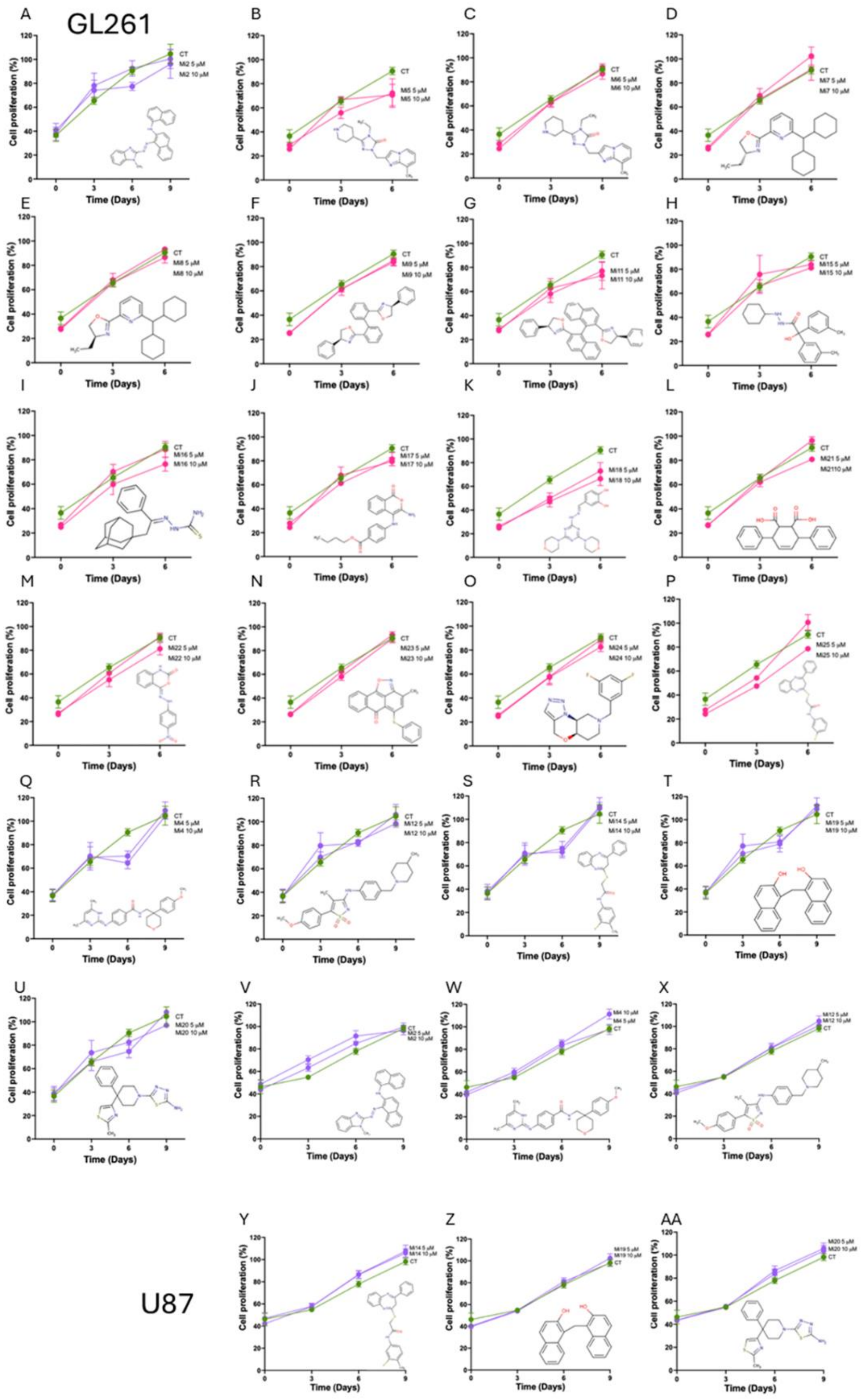

Figure S4: 25 compound screen results in GL261 and U87-MG A-U GL261 cells were exposed to 5 and 10 µM of each Mi compound. These graphs show the results of the 6- and 9-day screens of Mi2, Mi4-Mi24. V-AA shows the results of 9-day screens of U87-MG to Mi2, 4, 10, 12, 14, 19 and 20. None of these compounds inhibited GBM cell proliferation.

Table S1: Compounds assessed for direct binding to the human MIOS protein and GBM cell proliferation inhibition. Following a 4 million small molecule compound search to identify potential binding to human MIOS (NP_001357009.1), 25 were shortlisted and assigned a 'MIOS Inhibitor #' code Mi#. Molport ID are shown for each compound. Individual analysis of each compound for MIOS β-propeller region direct binding employed SwissDock, and the location of binding on the b-propeller region was described. Bioassays of each compound, to reduce cell proliferation of two GBM lines (GL261 and U87) were analysed (N/A not assessed). One compound, Mi3 (purple row) showed significant inhibition of cell proliferation in both cell lines, whilst Mi10 (green row) showed no inhibition of cell proliferation.

Mi	Molport ID	Predicted binding (ΔG)	Location of binding	GL261
1	Sigma- S21718-1EA	-10.76	Outside β-propeller	Reduction in proliferation
2	Molport-002-160-602	-9.12	Inside β-propeller	No effect
3	Molport-001-940-992	-9.04	Outside β-propeller	Reduction in proliferation
4	Molport-027-847-133	-8.77	Inside β-propeller	No effect
5	Molport-047-492-763	-8.64	Inside β-propeller	No effect
6	Molport-047-488-984	-7.30	Inside β-propeller	No effect
7	Molport-051-508-385	-8.06	Inside β-propeller	No effect
8	Molport-051-508-343	-8.13	Inside β-propeller	No effect
9	Molport-051-516-946	-8.24	Inside β-propeller	No effect
10	Molport-051-522-603	-9.11	Inside β-propeller	No effect
11	Molport-051-052-636	-9.12	Inside β-propeller	No effect
12	Molport-007-716-506	-9.14	Inside β-propeller	No effect
13	Molport-007-716-507	-8.66	Inside β-propeller	N/A
14	Molport-007-598-093	-8.13	Inside β-propeller	No effect
15	Molport-001-929-383	-8.11	Inside β-propeller	No effect
16	Molport-000-653-752	-7.90	Inside β-propeller	No effect
17	Molport-009-202-543	-7.83	Inside β-propeller	No effect
18	Molport-001-927-062	-12.16	Inside β-propeller	No effect
19	Molport-000-5250637	-7.20	Inside β-propeller	No effect
20	Molport-051-498-377	-7.75	Inside β-propeller	No effect
21	Molport-001-837-981	-7.61	Inside β-propeller	No effect
22	Molport-003-009-026	-7.70	Inside β-propeller	No effect
23	Molport-002-5560948	-7.57	Inside β-propeller	No effect
24	Molport-039-107-998	-7.50	Inside β-propeller	No effect
